# Ifi30 Is Required for Sprouting Angiogenesis During Caudal Vein Plexus Formation in Zebrafish

**DOI:** 10.3389/fphys.2022.919579

**Published:** 2022-07-13

**Authors:** Xiaoning Wang, Xiaojuan Ge, Yinyin Qin, Dong Liu, Changsheng Chen

**Affiliations:** School of Life Sciences, Nantong Laboratory of Development and Diseases, Key Laboratory of Neuroregeneration of Jiangsu and Ministry of Education, Co-innovation Center of Neuroregeneration, Nantong University, Nantong, China

**Keywords:** IFI30, zebrafish, sprouting angiogenesis, CVP formation, vascular development

## Abstract

Interferon-gamma-inducible protein 30 (IFI30) is a critical enzyme that mainly exists in immune cells and functions in reducing protein disulfide bonds in endocytosis-mediated protein degradation. Regardless of this, it is also found to be expressed in vascular system. However, the functions of IFI30 in vascular development remains unknown. Vascular network formation is a tightly controlled process coordinating a series of cell behaviors, including endothelial cell (EC) sprouting, proliferation, and anastomosis. In this work, we analyzed the function of zebrafish Ifi30, orthologous to the human IFI30, in vascular development during embryogenesis. The *ifi30* gene was found to be highly expressed in the caudal vein plexus (CVP) region of zebrafish embryos. Morpholino-mediated Ifi30 knockdown in zebrafish resulted in incomplete CVP formation with reduced loop numbers, area, and width. Further analyses implied that Ifi30 deficiency impaired cell behaviors of both ECs and macrophages, including cell proliferation and migration. Here, we demonstrate a novel role of IFI30, which was originally identified as a lysosomal thiol reductase involved in immune responses, in CVP development during embryogenesis. Our results suggest that Ifi30 is required for sprouting angiogenesis during CVP formation, which may offer an insight into the function of human IFI30 in angiogenesis under physiological or pathological conditions.

## Introduction

Interferon-gamma-inducible protein 30 (IFI30), also known as GILT (abbreviation for gamma-interferon–inducible lysosomal thiol reductase), is a key enzyme in antigen processing and presentation. It encodes a lysosomal thiol reductase that reduces protein disulfide bonds at low pH (Arunachalam et al., 2000). The enzyme localizes in endosomes or lysosomes and reduces protein disulfide bonds in endocytosed compartments for further degradation (Luster et al., 1988). IFI30 is constitutively expressed in professional antigen-presenting cells (APCs), including B cells, dendritic cells and monocytes/macrophages, and induced by interferon-γ (IFN-γ) in other cell types, such as tumor cells (Arunachalam et al., 1998; Maric et al., 2001; Lackman et al., 2007; Nguyen et al., 2016). In addition, *ifi30* has also been reported to be expressed in vascular niche and caudal hematopoietic tissue of zebrafish embryos, and involved in regulating hematopoietic stem and progenitor cells (HSPCs) expansion during embryogenesis (Cacialli et al., 2021). Although there is detectable expression of *ifi30* gene in zebrafish vascular system (Thisse and Thisse, 2004; Cacialli et al., 2021), whether it has a role in vascular development is unknown.

Here, we used zebrafish (*Danio Rerio*) as the model to study the role of Ifi30 in vascular development. The zebrafish model owns several unique advantages, e.g., the external development and optical transparency of zebrafish embryos allows high-resolution *in vivo* imaging of the blood vessel development, and the ease of genetic manipulation strategies like morpholino (MO) and CRISPR/Cas9 system facilitate the model establishment (Liu and Stainier, 2012; Irion et al., 2014; Moulton, 2017). Further, zebrafish Ifi30 is highly similar to its mammalian orthologs regrading to genomic organization as well as conserved domains (Cuiwei et al., 2012). In this work, we first performed reverse transcription (RT)-PCR experiment with isolated endothelial cells (ECs) from zebrafish embryos. The results demonstrated that *ifi30* is highly enriched in vascular ECs. Our *in situ* hybridization (ISH) analysis indicates that *ifi30* is expressed in the ventral region of the tail from 24 to 48 h post fertilization (hpf). All of which suggest a specific expression of *ifi30* in caudal vein plexus (CVP) and a potential role of Ifi30 in vascular development, especially for CVP formation. Loss-of-function of Ifi30 in zebrafish with an ATG-MO leads to defective CVP development, which can be partially rescued by *ifi30* mRNA injection. This result indicates its involvement in the development of vascular system in zebrafish. The incomplete CVP formation is proven to be a result of the impairment of EC behaviors by confocal imaging of control-MO and Ifi30-MO injected *Tg(fli1:nEGFP)* transgenic zebrafish line, in which the nuclei of ECs are fluorescently labeled by EGFP. Meanwhile, loss of Ifi30-caused developmental delay was excluded by further observation. As the CVP formation is mainly regulated by Bone morphogenetic protein 2 (Bmp2)-mediated angiogenesis (Wiley et al., 2011), we perform further ISH assay with Bmp2 signaling components. All these examined genes which are critical in this pathway exhibited down-regulated expression upon Ifi30 knockdown. Since IFI30 is constitutively expressed in macrophages and activated macrophages are also known to interact with ECs to promote vascular proliferation and vessel anastomosis (Lackman et al., 2007; Fantin et al., 2010; Gerri et al., 2017), we proposed that the deficiency in CVP formation might be caused by macrophage alteration. Confocal imaging of *Tg(coro1α:EGFP)* and ISH with the macrophage marker, *l-plastin*, exhibited a significant decrease in macrophage numbers in the region of CVP. All these results suggest a novel role Ifi30 in CVP formation in zebrafish.

## Materials and Methods

### Zebrafish and Ethics Statements

The wild-type AB line and transgenic lines *Tg(kdrl:EGFP)* (Wang et al., 2016), *Tg(fli1a:nEGFP)* (Wang et al., 2016), *Tg(kdrl:ras-mCherry)* (Chi et al., 2008), *Tg*(*fli1ep:EGFP-CAAX*)^*ntu666*^ (Chen et al. in prep.), and *Tg(coro1α:EGFP)* (Li et al., 2012), were used in this study. All zebrafish embryos and adult fishes were raised and maintained as previously described (Huang et al., 2013). All animal-related experiments were carried out following the NIH Guidelines for the care and use of laboratory animals (http://oacu.od.nih.gov/regs/index.htm), and animal protocols were ethically approved by the Administration Committee of Experimental Animals of Nantong University, Jiangsu Province, China (Approval ID: SYXK(SU) 20200711–001). Our study complied with the rules of the Guidelines for the care and use of laboratory animals (https://www.biomedcentral.com/getpublished/editorial-policies#standards+of+reporting). The study was carried out in compliance with the ARRIVE guidelines.

### FACS, RNA Extraction and RT-PCR

The *Tg(kdrl:EGFP)* Embryos were collected and manually dechorionated at 24 hpf, and followed by washing with PBC for three times and digested with 0.25% trypsin at 37°C. The digested cells were collected by centrifugation and allowed to pass through a 40 mm FACS tube (BD Falcon, 352340). The EGFP positive cells were sorted by fluorescence-activated cell sorting on FACS Aria3 (BD Biosciences). Afterwards, total RNA was extracted by TRIzolTM reagent (Thermo Fisher Scientific, 15596026) and reversely transcribed by using the HiScript III first Strand cDNA Synthesis Kit (Vazyme, R312-01). Apart from *ifi30*, two vascular markers (*kdrl* and *fli1a*), one arterial marker (*dll4*), and the housekeeping gene (*ef1a*), were also examined by RT-PCR. The primers for RT-PCR were listed in the Supplementary Table.

### *In situ* Hybridization (ISH)

ISH with antisense RNA probes was performed as described previously (Krueger et al., 2011). Templates for making probe to detect the expression of *ifi30*, *bmp2b*, *bmpr2a*, *dab2*, and *l-plastin*, were cloned from cDNA library, respectively. Primers for ISH were listed in Supplementary Table. Zebrafish embryos for ISH assay were collected and fixed with 4% paraformaldehyde (PFA) in PBS overnight. The fixed samples were dehydrated in gradient methanol solutions and then stored at−20°C for ISH assay. After hybridization, images of the embryos were acquired with an Olympus stereomicroscope MVX10 equipped with an Olympus DP71 camera.

### Morpholino

Translational blocking MO against Ifi30 and standard control MO were purchased from Gene Tools, LLC. The Ifi30 MO sequence was 5′-GGT​TAA​AGC​CGA​ACA​TGA​TGA​TTC​C-3′, and the standard MO sequence was 5′-CCT​CTT​ACC​TCA​GTT​ACA​ATT​TAT​A-3’. MOs were prepared according to the manufacturer’s instruction, and 2 nL of 0.3 mM MO oligo was microinjected per embryo at the one-cell stage in this study.

### mRNA Preparation and Injection

The coding sequence of *ifi30* and *mCherry* were subcloned into pCS2 + vector, respectively. The recombinant plasmids were linearized with Not I restriction enzyme (NEB, R3189), and transcribed using the mMESSAGE mMACHIN Kit (Thermo Fisher Scientific, AM1340). For rescue and overexpression experiments, 2 nl *ifi30* and *mCherry* mRNA mixture (1:1) was either co-injected with Ifi30-MO or injected alone into one-cell stage embryos. The injected mRNA concentration was 100 ng/ml.

### Confocal Imaging and Quantitative Analysis

For confocal imaging, zebrafish embryos treated with 1-phenyl-2-thiourea (Sigma Aldrich) to inhibit the pigmentation. After manually dechorionation, embryos were anesthetized with egg water/0.16 mg/ml tricaine/1% one-phenyl-2-thiourea (Sigma) and embedded in 0.6% low melting agarose. Living imaging was performed with Nikon A1R confocal microscopy. The morphology of the CVP including loop numbers, area, width, and vessel diameter, were measured with ImageJ software. The CVP width and VV diameter were measured at three different locations. The average of these data points was statistically used as the diameter of the analyzed vessel.

### Statistical Analysis

Statistical analysis was performed with *t*-test or One-Way ANOVA. All data is presented as mean ± *s*. e.m, and *p* < 0.05 was considered to be statistically significant.

## Results

### Interferon-Gamma-Inducible Protein 30 Is Highly Enriched in ECs and Preferentially Expressed in Caudal Vein Plexus During Zebrafish Embryogenesis

**FIGURE 1:**
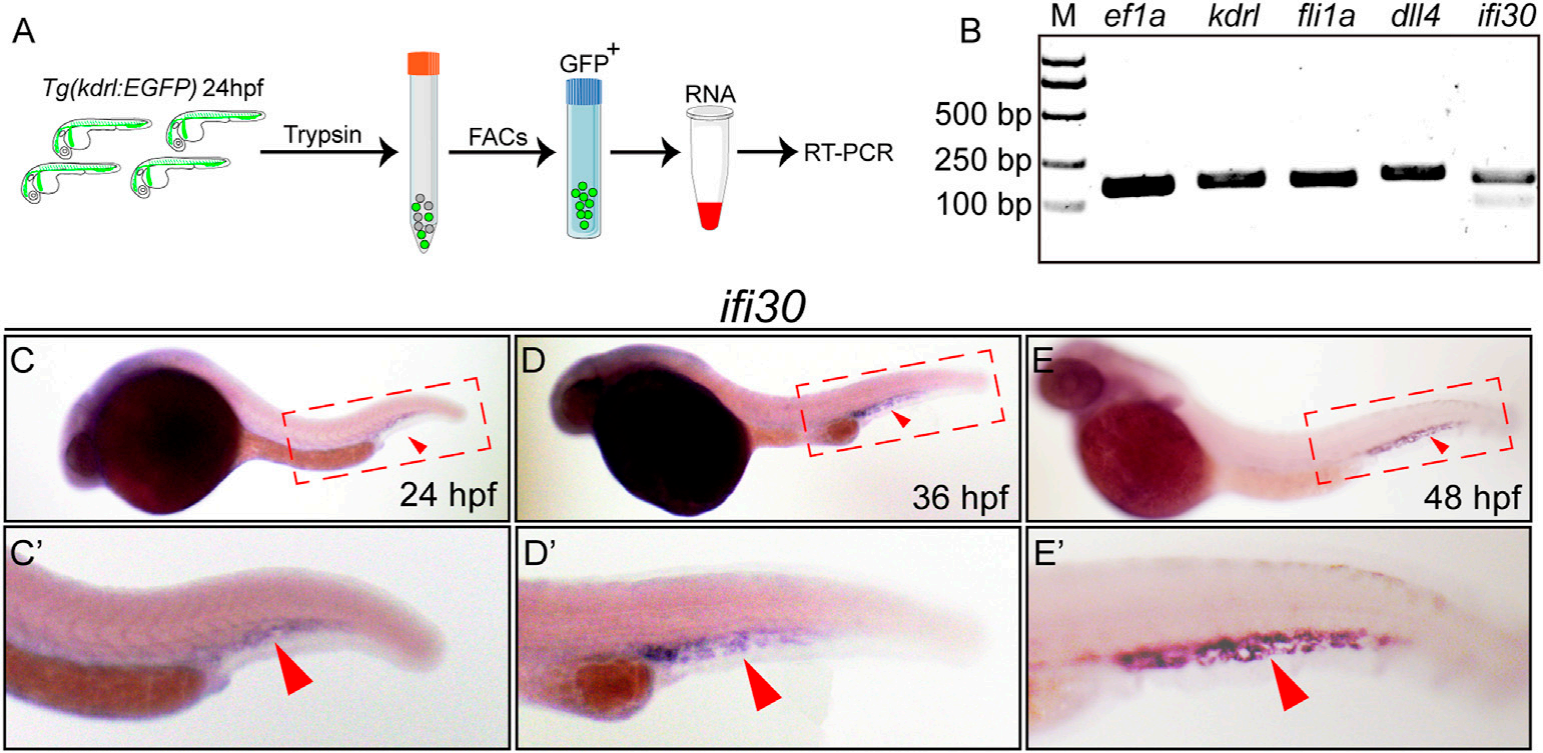
ifi30 enriches in endothelial cells (ECs) and expresses in caudal vein plexus (CVP) region **(A)** Schematic representation of isolation of ECs from *Tg(kdrl:EGFP)* zebrafish embryos for RNA extraction and RT-PCR **(B)** Expressions of *kdrl*, *fli1a*, *dll4*, and *ifi30* in ECs are examined by RT-PCR. *ef1a* is used as the housekeeping gene. M indicates the DNA ladder and the corresponding sizes are marked on the left **(C–E)** Expression of *ifi30* in zebrafish embryos from 24 to 48 hpf **(C′-E′)** Magnifications of the red dotted boxes in the corresponding **(C) (D)**, and **(E)** panel, respectively. *Ifi30* is specially expressed in the CVP region (indicated with red arrowhead).

To characterize whether *ifi30* is an EC-expressed factor, we performed RT-PCR experiment with isolated ECs from *Tg(kdrl:EGFP)* zebrafish embryos (Figure 1A). The expression *ifi30* could be detected in vascular ECs, which was consistent with the expression of known EC-specific genes, such as *kdrl*, *fli1a*, and *dll4* (Figure 1B). Next, we performed whole-mount *in situ* hybridization (WISH) to examine its spatiotemporal expression in developing embryos. The *ifi30* expression was mainly restricted to the tail region, which corresponds to the location of CVP (Figures 1C–E). The *ifi30* mRNA transcripts was detectable at 24 hpf, which was just prior to the onset of CVP development [approx. 25 hpf (Wiley et al., 2011)], and its expression was increased and reached the highest level at 48 hpf, which is a time point for CVP remodeling (Wisniewski et al., 2020) (Figures 1C′–E′). All these results suggest a potential role of Ifi30 in vascular development and remodeling.

### Interferon-Gamma-Inducible Protein 30 Loss-Of-Function Impairs Caudal Vein Plexus Formation

**FIGURE 2:**
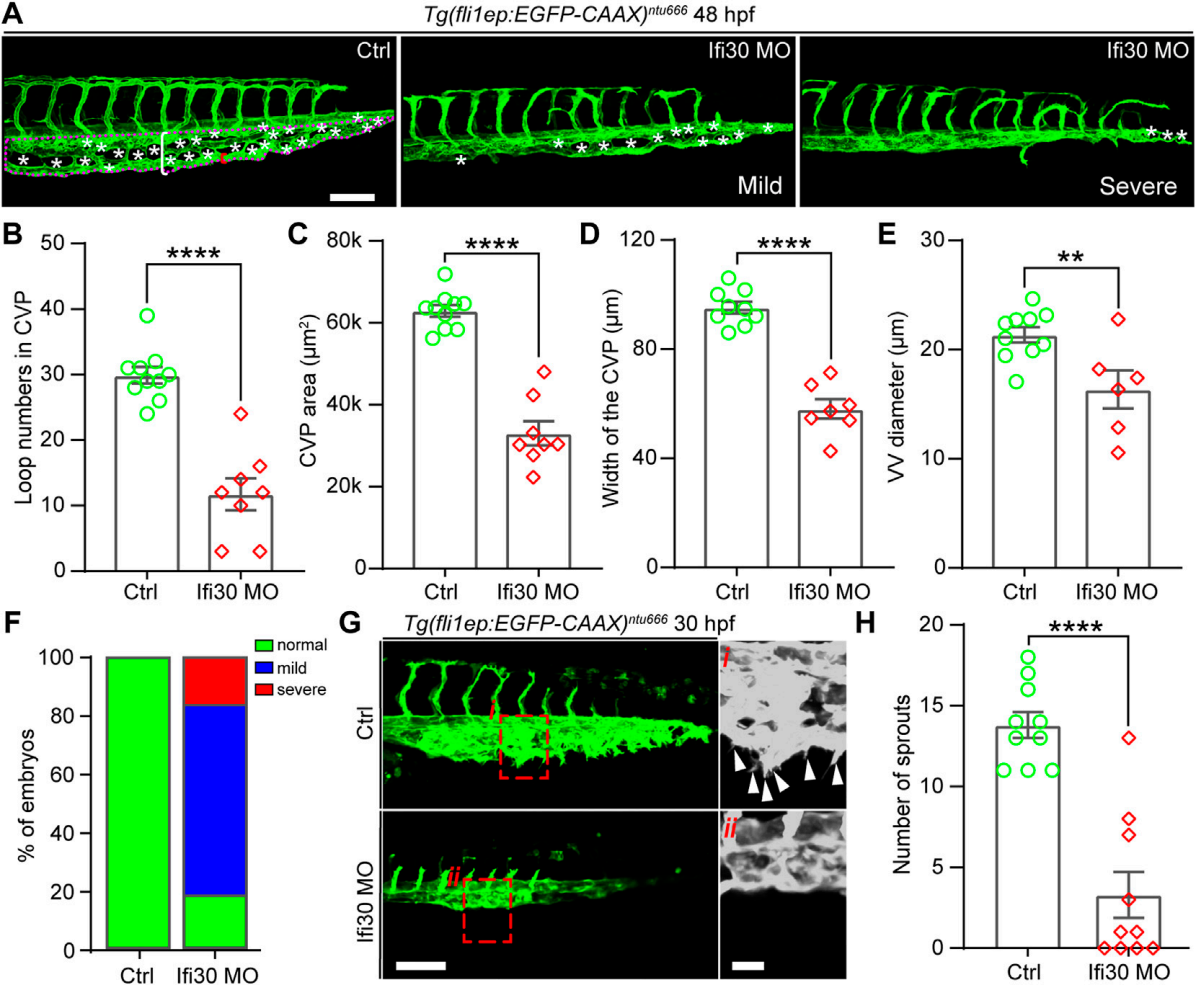
Ifi30 knockdown leads to defective CVP morphogenesis in zebrafish embryos **(A)** Confocal images of CVP phenotypes in 48-hpf *Tg*(*fli1ep:EGFP-CAAX*)^*ntu666*^ control embryos and embryos injected with Ifi30 morpholino (MO). The aberrant CVP phenotypes are defined as mild and severe type, respectively **(B–E)** Quantification of CVP loop numbers (asterisks), total CVP area (magenta dotted line outlined area), CVP width (white square bracket), and ventral vein (VV) diameter (red square bracket) in control and Ifi30 morphants **(F)** The incidence of normal and defective CVP phenotypes in control *Tg*(*fli1ep:EGFP-CAAX*)^*ntu666*^ embryos (n = 41) and Ifi30 morphant embryos (n = 40) **(G)** Confocal images of CVP region in control and Ifi30 morphants at 30 hpf. Right panels are the magnifications of the red dotted boxes. Arrowheads indicate the venous sprouts **(H)** Quantification of venous sprouts of CVP. Error bars represent SEM. **, *p* < 0.01. ****, *p* < 0.0001. Scale bars, 100 µm.

To investigate the function of Ifi30 in vascular development, a translational blocking morpholino (MO)-mediated knockdown was utilized to downregulate Ifi30 expression in the transgenic line *Tg*(*fli1ep:EGFP-CAAX*)^*ntu666*^ (Chen et al. in prep.), in which a chimera endothelial enhancer/promoter fragment (fli1ep) was employed to drive the specific expression of EGFP in ECs, and the CAAX membrane targeting motif enabled the localization of EGFP at the EC surface. The efficiency of Ifi30 MO was validated in zebrafish embryos (Supplementary Figure S1A and Supplementary Figure S1B). Compared with control MO injected embryos, the Ifi30 morphants displayed defective CVP morphology at 48 hpf, including reduced loop numbers in the CVP, decreased CVP area and width, and narrowed ventral vein (VV) (Figures 2A–E). Most morphants showed mild phenotypes with an incomplete honeycomb structure, whereas the severe phenotypes even exhibited the loss of honey-comb structure in the CVP (Figure 2A-right panel). More than 80% of the morphants showed defective CVP morphology and among them the rates of mild (incomplete honeycomb) and severe (without honeycomb structure) phenotypes were around 75% and 25% respectively (Figure 2F). As Ifi30 was involved in CVP formation during vascular development, we supposed that it might exert a role in regulating endothelial tip cell behaviors. To test this, we performed live imaging and time-lapse imaging with *Tg*(*fli1ep:EGFP-CAAX*)^*ntu666*^ embryos. Compared to control, Ifi30 morphants displayed almost complete loss of venous sprouts (Figures 2G,H, Supplementary Figure S2, and Supplementary Movie S1 and Supplementary Movie S2), suggesting the suppression of EC migration by Ifi30 knockdown.

### Interferon-Gamma-Inducible Protein 30 mRNA Injection Rescues the Phenotype Induced by Ifi30 Knockdown

**FIGURE 3:**
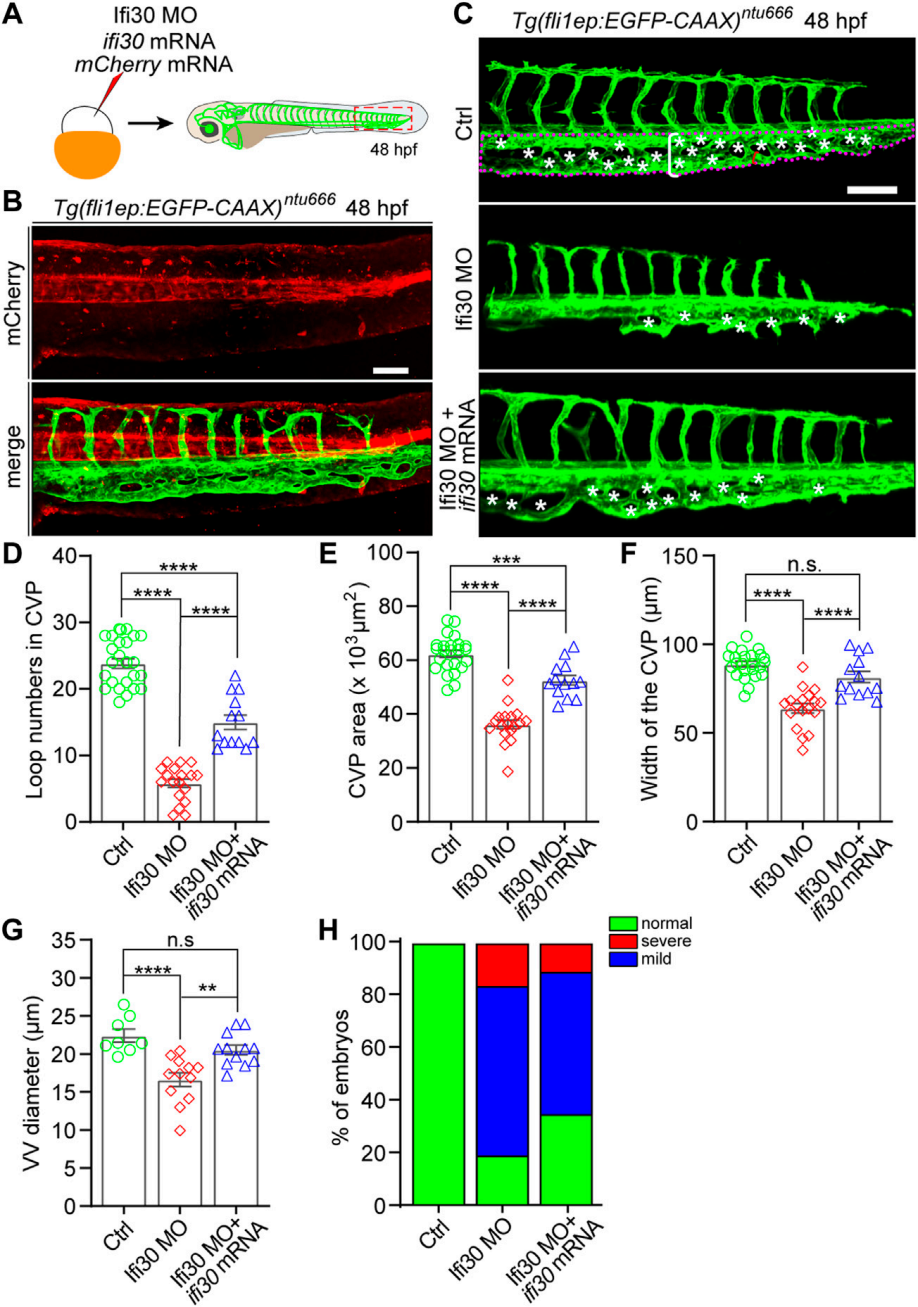
Ifi30 overexpression rescues the defective CVP phenotypes of Ifi30 morphants **(A)** Schematic representation of the rescue experiment. The mixture of Ifi30 MO, *ifi30* mRNA, and mCherry mRNA is injected into one-cell stage *Tg*(*fli1ep:EGFP-CAAX*)^*ntu666*^ embryos. The mCherry mRNA is used to verify the working efficiency of mRNA injection by examining the red fluorescence post injection **(B) (C–G)** The CVP loop numbers (asterisks), total CVP area (white dotted line outlined area), CVP width (white square bracket), and ventral vein (VV) diameter (red square bracket) in Ifi30 morphants are all rescued by *ifi30* mRNA injection **(H)** The incidence of normal and defective CVP phenotypes in control *Tg*(*fli1ep:EGFP-CAAX*)^*ntu666*^ embryos (n = 34), embryos injected with Ifi30 MO (n = 41), and embryos co-injected with Ifi30 MO and *ifi30* mRNA (n = 34). Error bars represent SEM. n. s., not significant. **, *p* < 0.01. ***, *p* < 0.001. ****, *p* < 0.0001. Scale bars, 100 µm.

To examine whether the phenotypes of Ifi30 morphants were caused by Ifi30 knockdown, we performed a rescue experiment by co-injecting *ifi30* mRNA with Ifi30 MO into one-cell-stage of *Tg*(*fli1ep:EGFP-CAAX*)^*ntu666*^ zebrafish embryos (Figure 3A). The *ifi30* mRNA containing 15 bp mismatches in the coding region was not translationally blocked by the Ifi30 MO (Supplementary Figure S1C and Supplementary Figure S1D). The mRNA of *mCherry* was also co-injected as an indicator (Figure 3B). The defective CVP morphology in the morphants was partially rescued by *ifi30* mRNA co-injection, such as the increases in loop numbers, CVP areas and widths, VV diameters, and the formation of complete honey-comb structure in CVP (Figures 3C–G). Meanwhile, the rates of both mild and severe phenotypes were decreased (Figure 3H). Although all these aforementioned parameters could not be recovered to control embryos, it is sufficient to prove the role of Ifi30 in vascular development, especially in CVP formation. However, injection of *ifi30* mRNA alone into *Tg(kdrl:EGFP)* embryos to overexpress *ifi30* had no obvious effects on vascular development, like ectopic sprouting or hyperbranching, when compared to control MO injected embryos (Supplementary Figure S3). All these results suggest that Ifi30 is required for CVP development in zebrafish.

### Interferon-Gamma-Inducible Protein 30 Deficiency Impairs EC Proliferation During Caudal Vein Plexus Formation

**FIGURE 4:**
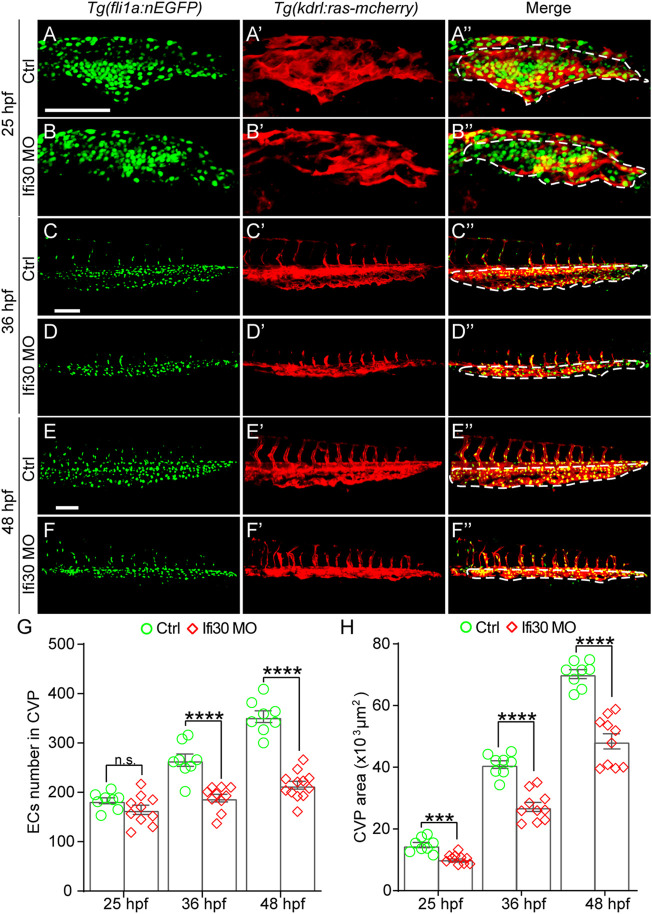
Ifi30 loss-of-function impairs endothelial cell (EC) proliferation during CVP development **(A–F")** Confocal images of *Tg(kdrl:ras-mCherry);Tg(fli1:nEGFP)* double transgenic embryos and embryos injected with Ifi30 MO at 25 hpf **(A,B")**, 36 hpf **(C,D")**, and 48 hpf **(E,F")**. The EC numbers in the CVP region and CVP area (white dotted line outlined area) were quantified **(G,H)**. Error bars represent SEM. n. s., not significant. ***, *p* < 0.001. ****, *p* < 0.0001. Scale bars, 100 µm.

Loss of Ifi30 leads to CVP growth defects, suggesting a possible disruption of EC proliferation. To determine whether the cell proliferation is affected by Ifi30 dysfunction, we injected Ifi30-MO into the *Tg(kdrl:ras-mCherry);Tg(fli1a:nEGFP)* double transgenic line, which allows the visualization of the EGFP-tagged EC nuclei and mCherry-tagged vascular ECs, respectively (Figure 4A–F″). The number of ECs was not affected by Ifi30 loss-of-function at the onset of CVP sprouting (25 hpf) (Figure 4G). However, the EC numbers were much less in Ifi30 morphants at 36 and 48 hpf when compared to control-MO injected embryos (Figure 4G). Moreover, no apoptotic ECs were detected in the CVP region (Supplementary Figure S4). These data suggests a role of Ifi30 in regulating EC proliferation in CVP. Although there was no difference in EC numbers between control and Ifi30 morphants prior to CVP formation (25 hpf), Ifi30 morphants had smaller CVP size (Figure 4H), suggesting the interruption of cell migration by Ifi30 deficiency.

### Interferon-Gamma-Inducible Protein 30 Knockdown Impairs Bmp2 Signaling in Zebrafish

**FIGURE 5:**
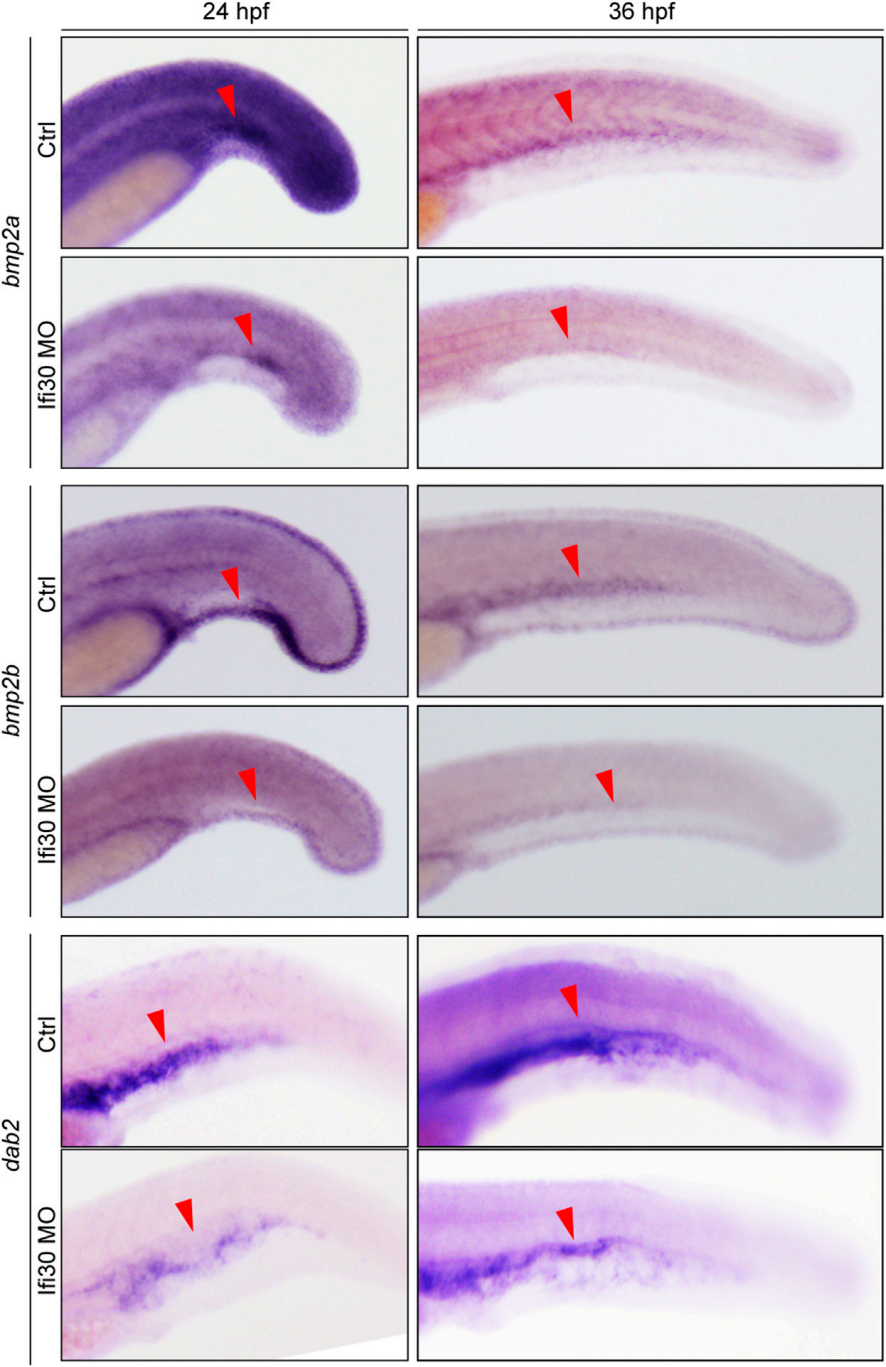
Ifi30 knockdown disrupts Bmp2 signaling pathway. *In situ* hybridization for *bmp2b*, *bmpr2a*, and *dab2* of control embryos and embryos injected with Ifi30 MO at 24 and 36 hpf.

As previously reported, Bmp2 signaling pathway is critical for CVP development during zebrafish embryogenesis (Wiley et al., 2011). Also, the Disabled homolog 2 (Dab2), a cargo-specific adaptor protein for Clathrin, is involved in CVP formation in zebrafish as an essential modulator of Bmp2 signaling (Kim et al., 2012). To test if the Bmp2 signaling pathway is altered upon Ifi30 loss-of-function, we performed ISH assay with the ligand *bmp2b*, the receptor *bmpr2a*, as well as *dab2*. ISH analysis showed that the expressions of all these three Bmp2 signaling components were significantly decreased in Ifi30 morphants (Figure 5). These results suggest that the Bmp2 signaling pathway was disrupted upon Ifi30 loss-of-function.

### Interferon-Gamma-Inducible Protein 30 Knockdown Leads to Macrophage Reduction During Caudal Vein Plexus Development

**FIGURE 6:**
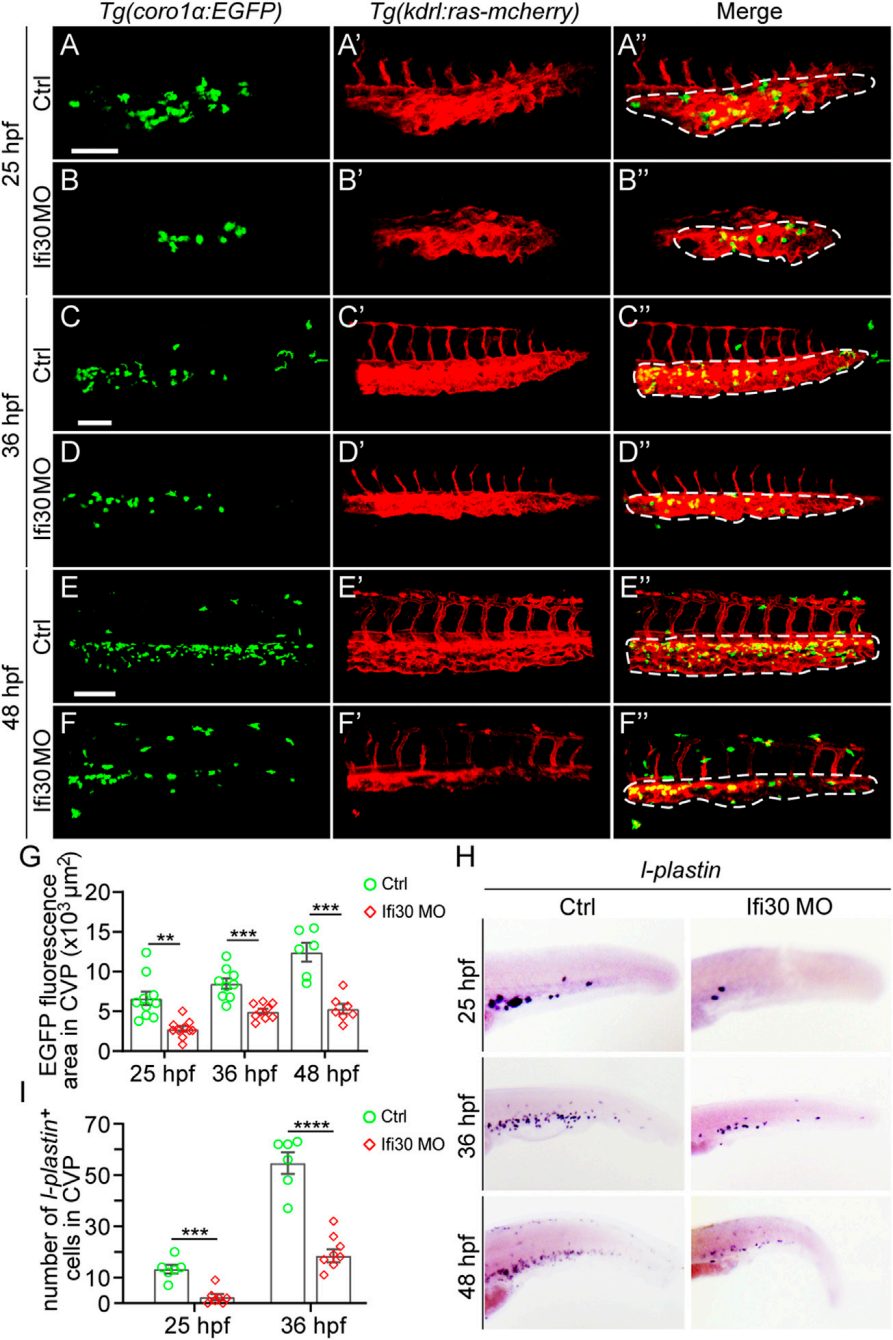
Macrophages are reduced upon Ifi30 knockdown **(A–F")** Confocal images of *Tg(kdrl:ras-mCherry);Tg(coro1a:EGFP)* double transgenic embryos and embryos injected with Ifi30 MO at 25 hpf **(A,B")**, 36 hpf **(C,D")**, and 48 hpf **(E,F") (G)** Fluorescence intensities in the CVP region (dashed line outlined area) of GFP channel are quantified with ImageJ **(H)** *In situ* hybridization (ISH) assay of *l-plastin* (macrophage marker) in wild-type (WT) and Ifi30 morphant embryos at 25, 36, and 48 hpf, respectively. The number of *l-plastin*-positive cells in the CVP region (dashed line outlined area) is counted and compared between WT and Ifi30 morphants **(I)**. Error bars represent SEM. **, *p* < 0.01. ***, *p* < 0.001. ****, *p* < 0.0001. Scale bars, 100 µm.

In previous studies, macrophages have been reported to play a role in developmental angiogenesis (Fantin et al., 2010; Gerri et al., 2017). It is still unclear whether macrophages are altered in Ifi30 morphants. To examine this, we first injected Ifi30 MO into *Tg(kdrl:ras-mCherry);Tg(coro1α:EGFP)* double transgenic line, where ECs were labeled with mCherry and EGFP was expressed in both macrophages and neutrophils (Li et al., 2012; Liu et al., 2016). The fluorescence size of EGFP-positive immune cells was significantly reduced in the CVP area at 25, 36, and 48 hpf in Ifi30-deficient embryos (Figure 6). To further determine if the number of macrophages decreased, we performed ISH assay with *l-plastin*. Although *l-plastin* is a pan-leukocyte marker, it is mostly expressed in macrophages, especially at the early developmental stage in zebrafish (Herbomel et al., 1999; Krueger et al., 2011; Petrie et al., 2014). In Ifi30 morphants, *l-plastin* had a lower expression in CVP region when compared to control-MO injected siblings. We then manually counted the *l-plastin*-expressed macrophages in CVP area and found that Ifi30 deficiency caused a significant decrease in macrophage numbers (Figure 6). The results suggested that the decreased macrophages might contribute to the defective CVP phenotype of Ifi30 morphants.

## Discussion

IFI30 is a lysosomal thiol reductase expressed constitutively in antigen-presenting cells and can be induced by IFN-γ in other cell types (Arunachalam et al., 2000). It exerts a function in processing antigen proteins for major histocompatibility complex (MHC) class II-restricted presentation to CD4-positive T lymphocytes by reducing the disulfide bonds of phagocytosed antigenic proteins in the endocytic compartments (Maric et al., 2001). Apart from its involvement in immune responses, whether IFI30 has a role in other aspects like angiogenesis is lacking although its expression is detected in the vascular system (Cacialli et al., 2021). Surprisingly, we found that knockdown of Ifi30 in zebrafish resulted in defective vascular plexus formation, and the developmental deficiency could be rescued by Ifi30 overexpression. However, overexpressing Ifi30 in control embryos did not produce obvious effects on the establishment of the whole vascular system in zebrafish. To confirm the role of Ifi30 in CVP formation, we generated the *ifi30*-knockout mutant using CRISPR/Cas9 genome editing strategy with *Tg*(*fli1ep:EGFP-CAAX*)^*ntu666*^ transgenic zebrafish (Supplementary Figure S5). The G0 generation chimeras also recapitulated the vascular phenotypes of Ifi30 morphants (Supplementary Figure S5). Moreover, the defective vasculature caused by loss of Ifi30 was not a result of developmental delay. In addition to defective CVP development, we also noticed that the intersegmental vessel (ISV) sprouting and dorsal longitudinal anastomotic vessel (DLAV) formation were slightly affected by Ifi30 loss-of-function, such as vessel rupture and narrowing (Supplementary Figure S6). There might be several reasons behind this and further investigation is needed to decipher them. In addition, Ifi30 morphants exhibited pericardium edema at the later developmental stage, further indicating the damage to the vascular system (Supplementary Figure S7). Based on these observations, we inferred that Ifi30 is required for vascular development.

To gain further insight into the cellular mechanism underlying the regulation of CVP formation by Ifi30, we monitored the EC behaviors during CVP formation upon Ifi30 knockdown. At the early stage of CVP formation, the number of ventral sprouts in endothelial tip cells were significantly reduced in Ifi30 MO-injected embryos, suggesting the involvement of Ifi30 in regulating cell migration. Additionally, the number of ECs was also remarkably decreased in Ifi30 morphants during CVP development, indicating that EC proliferation was perturbed by the loss of Ifi30. As Bmp2 signaling pathway is critical for the formation of vascular networks in zebrafish (Wiley et al., 2011; Kim et al., 2012; Weijts et al., 2021), we further checked the expression of Bmp2 pathway components by ISH assay. The expressions of the ligand *bmp2b* and the receptor *bmpr2a* were barely detected in Ifi30 morphants. As a key modulator of Bmp2 signaling, *dab2* also displayed a reduced size of the expression domain in CVP area. These results suggest that Bmp2 signaling pathway may involve in Ifi30-mediated CVP formation. Further exploration is required to decipher the underlying mechanism.

Although IFI30 mainly participates in antigen processing and immune responses, it is also highly associated with tumor progression and angiogenesis (Liu et al., 2020). A myriad of studies have reported that macrophages could interact with ECs to regulate angiogenesis *via* inducing vascular proliferation, and/or promoting vessel anastomosis (Polverini et al., 1977; Leibovich et al., 1987; Fantin et al., 2010; Ligresti et al., 2011; Squadrito and De Palma, 2011; Gerri et al., 2017). As a macrophage-expressed factor, the idea that Ifi30 modulates CVP formation in zebrafish *via* macrophages is proposed. Our results demonstrated that Ifi30 deficiency led to the reduction of macrophages, which may result in the impaired CVP development subsequently. All these findings suggested that the incomplete CVP structure might have relations with macrophages reduction. Activated macrophages are often characterized as the enrichment of intracellular vacuoles and pinocytotic vesicles (Polverini et al., 1977), and IFI30 is a key component and the only enzyme known to catalyze disulfide bond reduction in the endocytic pathway (Maric et al., 2001; Balce et al., 2014; Li et al., 2021). Therefore, further exploring the role of IFI30 in vascular development associated with macrophages, and deciphering the mechanism underlying the vascular regulation of by IFI30 are still needed. In addition, IFI30 is found to be upregulated in some metastatic tumor types, such as melanoma and glioma (Nguyen et al., 2016; Liu et al., 2020). These malignancies are generally characterized by a high metastatic trait and strongly correlated with neovascularization, suggesting a potential role of IFI30 in promoting tumor angiogenesis and cell proliferation. Therefore, IFI30 can be used as a diagnostic and prognostic biomarker in cancers. Overall, our data indicates that Ifi30 is required for the formation of CVP in zebrafish and involved in sprouting angiogenesis through regulating EC and macrophage behaviors. The present study gives a new insight into the role of IFI30 in vascular development and provides potential strategies for cancer treatment.

## Data Availability

The original contributions presented in the study are included in the article/Supplementary Materials, further inquiries can be directed to the corresponding authors.
