# Supplementary material for: Ifi30 Is Required for Sprouting Angiogenesis During Caudal Vein Plexus Formation in Zebrafish
Source: Front Physiol. 2022 Jul 13;13:919579. doi: 10.3389/fphys.2022.919579 (PMC9325957; doi:10.3389/fphys.2022.919579)
Supplement: Supplementary file 1 [file Table1.DOCX]

Supplementary Material

# Supplementary Methods

**TUNEL assay**

For TUNEL assay, zebrafish embryos at 30 hpf were fixed in 4% PFA at 4°C overnight. After being rinsed with PBST for 10 minutes, the embryos were incubated in proteinase K for 2 minutes, followed by twice PBST washes for 5 minutes each time. Then the embryos were refixed with 4% PFA for 10 minutes and rinsed with PBST for three times. TUNEL staining was carried out ﻿according to the manufacturer’s instructions (Cell death detection kit, Roche).

**CRISPR/Cas9-mediated gene editing of *ifi30* in zebrafish**

A CRISPR/Cas9-mediated approach was used to generate *ifi30*-deleted mutants. The target site of CRISPR/Cas9 designed to identify the sequence in the 1^st^ exon of *ifi30* was 5’-GGACATAGCAGCTGAATGTGGGG-3’(underlined was the PAM sequence). For the sgRNA synthesis, a forward primer (5’- TAATACGACTCACTATAGGACATAGCAGCTGAATGTGGTTTTAGAGCTAGAAATAGC -3’) containing a T7 promoter region and an *ifi30* gene-targeting region, and a universal reverse primer (5’-AAAAAAAGCACCGACTCGGTGCCAC-3’) were used in the PCR amplification with pT7 plasmid as the template to obtain the sgDNA, which was then transcribed into sgRNA in vitro using the T7 mMessage mMachine kit (Ambion). Micro-injection was performed with 1-2 cell stage zebrafish embryos, and each embryo was co-injected with 100 pg sgRNA and 200 pg Cas9 mRNA. G0 generations were examined by PCR, followed by Sanger sequencing.

## Supplementary Figures


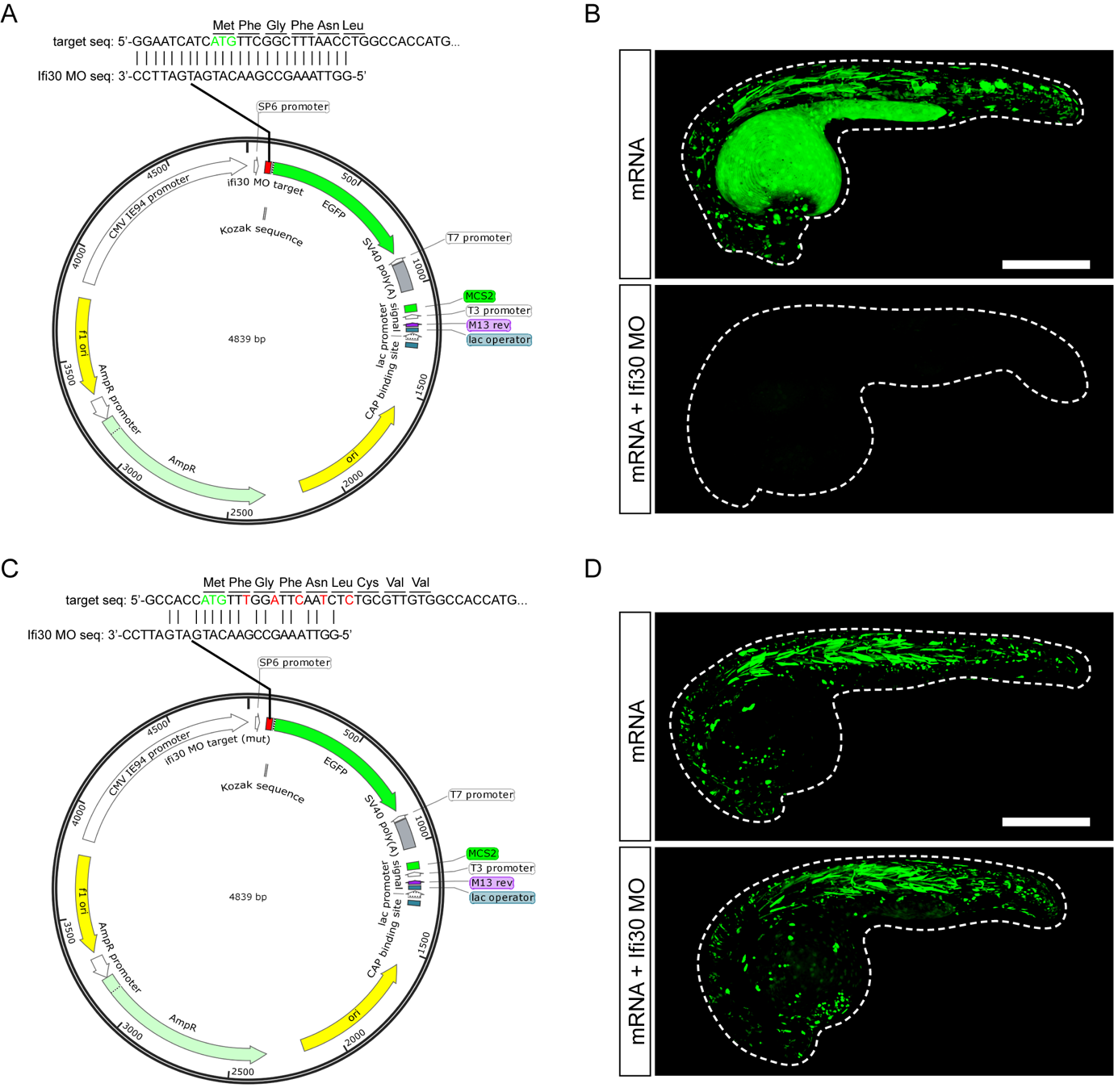


**Supplementary Figure 1.** ﻿**Assays of translation-blocking activity of Ifi30 MO. (A)** The MO targeting sequence is subcloned into pCS2+ vector prior to *EGFP* coding sequence. The recombinant plasmid is linearized and transcribed into mRNA in vitro using SP6 polymerase. **(B)** The mRNA is either injected alone (upper) or co-injected with Ifi30 MO (lower) into zebrafish embryos. The MO can effectively block the translation of EGFP. **(C)** The first 25 bp of silent mutated *ifi30* coding sequence is subcloned into pCS2+ vector prior to *EGFP* coding sequence. The recombinant plasmid is linearized and transcribed into mRNA in vitro using SP6 polymerase. **(D)** The mRNA is either injected alone (upper) or co-injected with Ifi30 MO (lower) into zebrafish embryos. The EGFP expression (lower) indicates that the MO is incapable of blocking the ifi30 mRNA translation in the following rescue experiment. Scale bars, 400 μm.


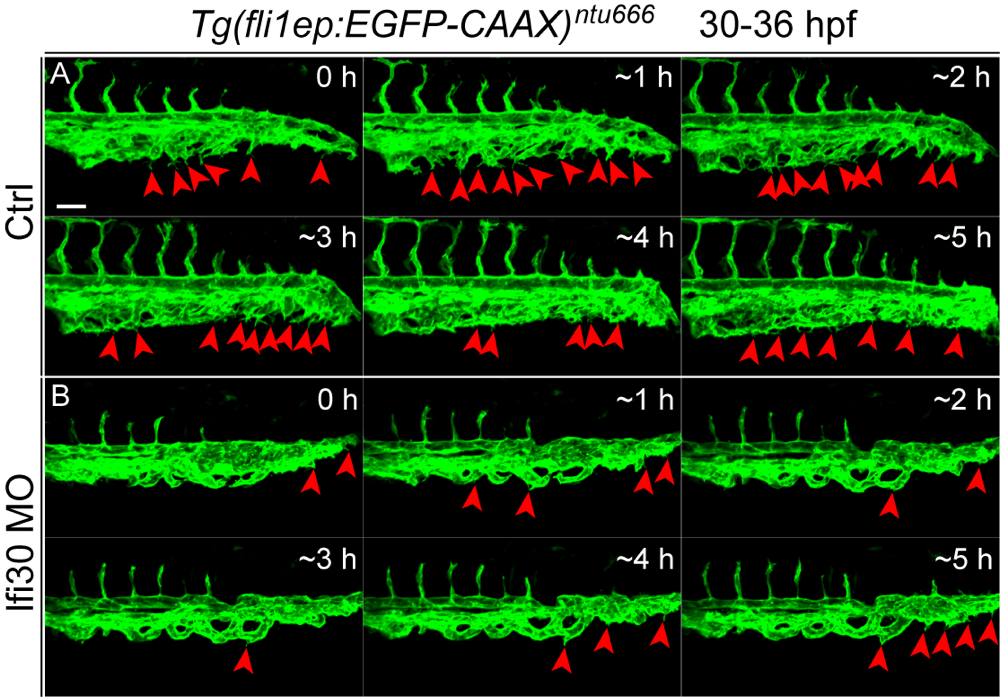


**Supplementary Figure 2. Ifi30 knockdown impairs ventral sprouting and EC migration in CVP.** Time-lapse images of CVP development in *Tg(fli1ep:EGFP-CAAX)^ntu666^* transgenic embryos, and embryos injected with Ifi30 MO from 30 to 36 hpf. Red arrowheads indicate the ventral sprouts. Scale bar, 50 µm.


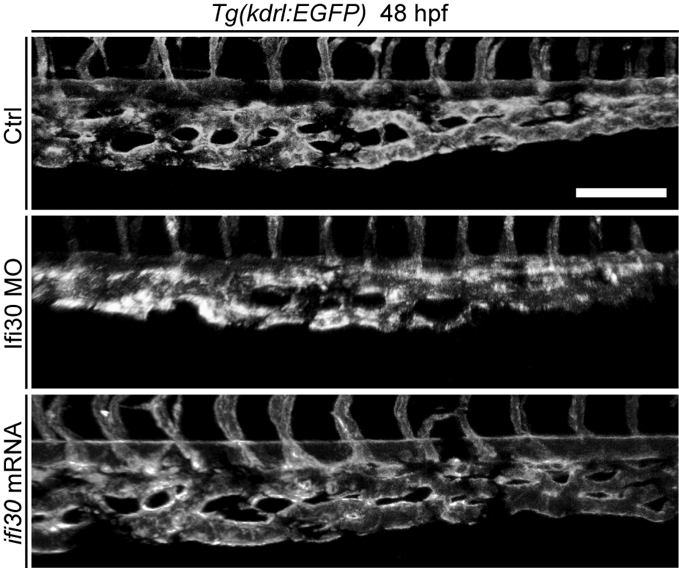


**Supplementary Figure 3.** **The CVP formation is not affected by *ifi30* mRNA injection alone.** Confocal images of CVP structures in *Tg(kdrl:EGFP)* transgenic embryos, and embryos injected with Ifi30 MO, and embryos injected with *ifi30* mRNA at 48 hpf. Scale bar, 100 µm.


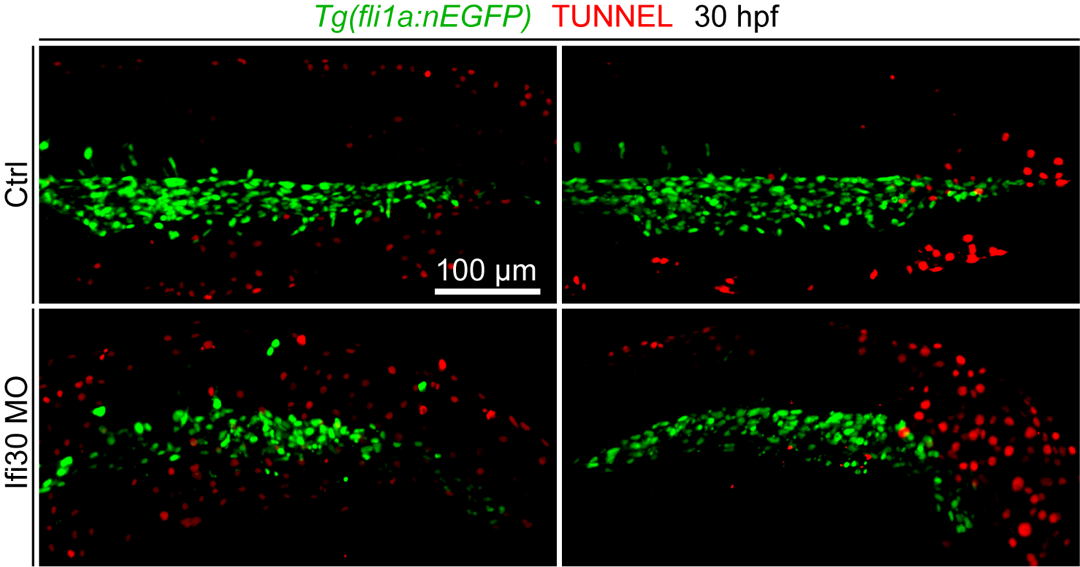


**Supplementary Figure 4.** **Ifi30 knockdown has no effects on EC apoptosis during CVP development.** TUNEL assay of ECs in CVP region in *Tg(kdrl:EGFP)* transgenic embryos, and embryos injected with Ifi30 MO at 30 hpf. ﻿The apoptotic cells were only detected on the skin and in the epidermis in the tail region (red fluorescence signal). No apoptotic cells were observed to be colocalized with ECs. Scale bar, 100 µm.

**
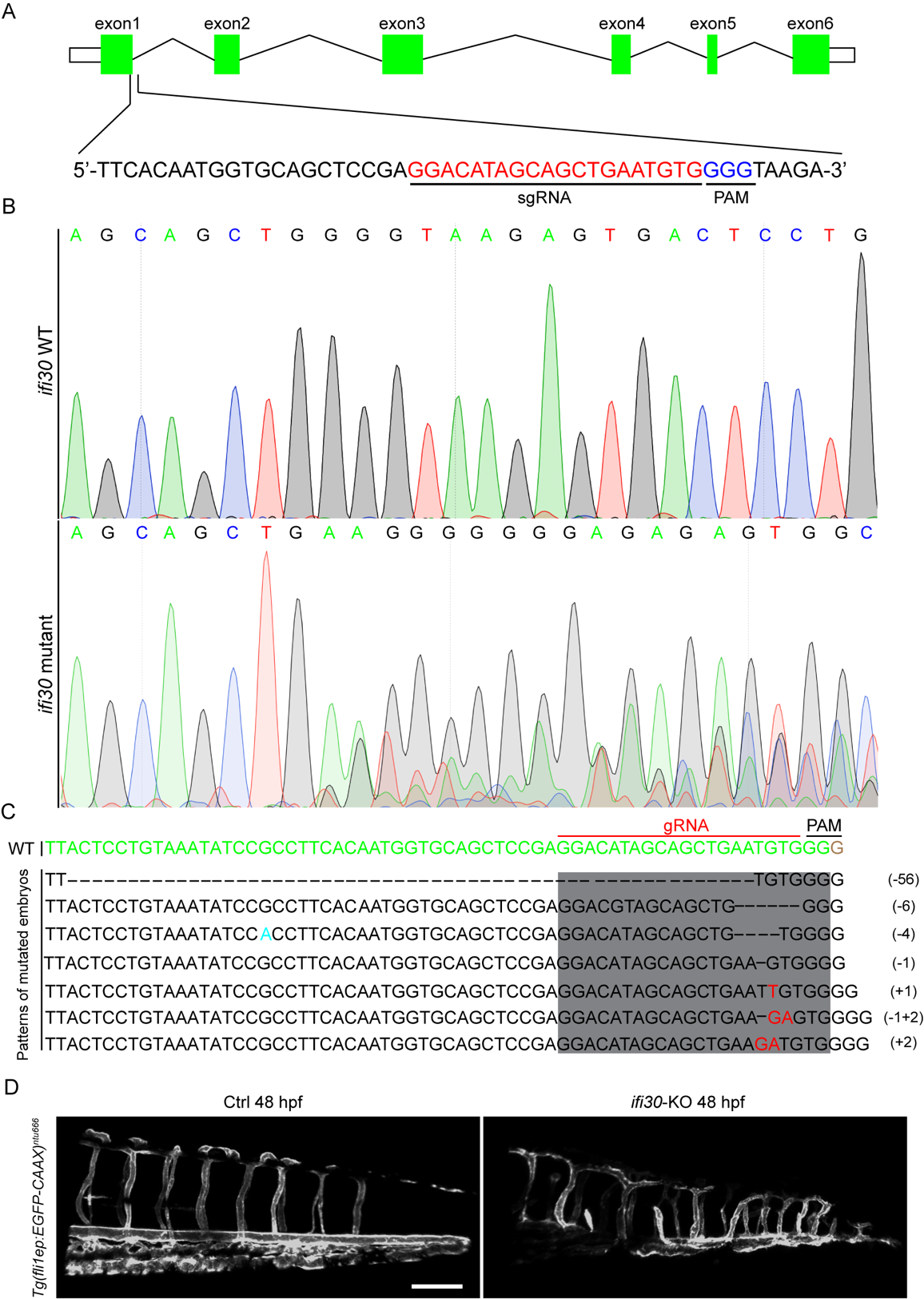
**

**Supplementary Figure 5.** **CRISPR/Cas9-mediated *ifi30* knockout in** ***Tg(fli1ep:EGFP-CAAX)^ntu666^* line. (A)** The designed single-guide RNA (sgRNA) is targeting the sequence at the end of the 1^st^ exon in *ifi30*. **(B)** Various mutated patterns were obtained. **(C)** *ifi30* mutants exhibited defective CVP development. Scale bar, 100 µm.


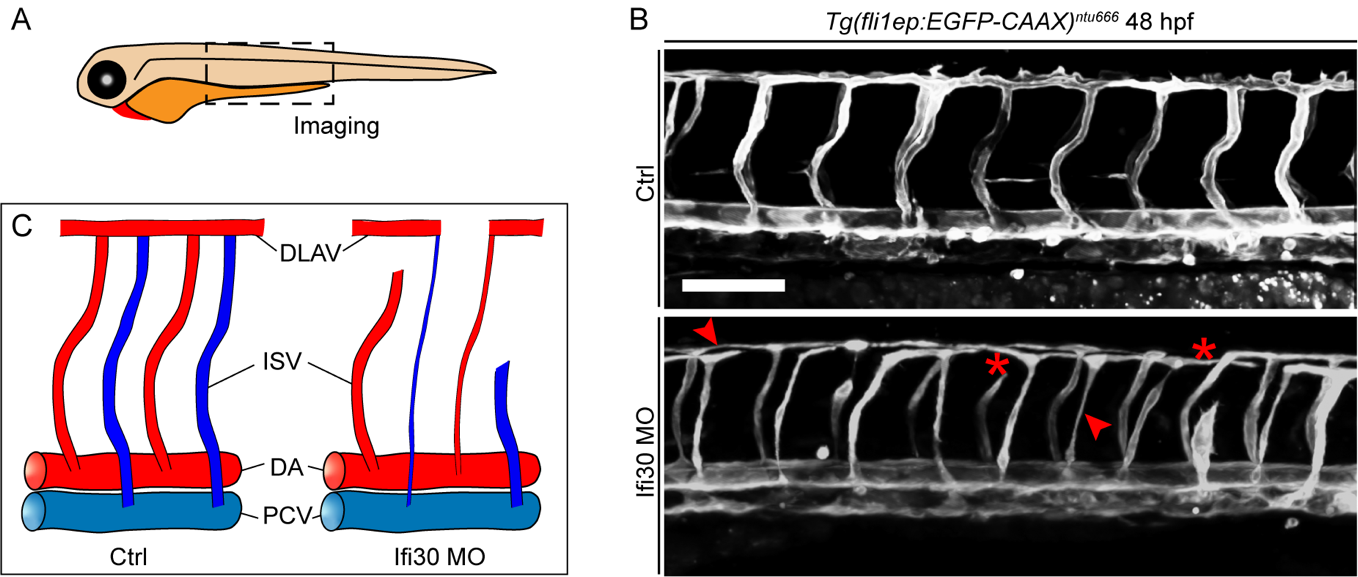


**Supplementary Figure 6.** **Loss of Ifi30 leads to abnormal ISV and DLAV development. (A)** The trunk vessels of zebrafish were imaged. **(B)** Ifi30 morphants show narrowed or ruptured ISVs and DLAV. Arrowheads and asterisks indicate narrowed and ruptured vessels, respectively. Scale bar, 100 µm. **(C)** Schematic representation of defective phenotype caused by Ifi30 knockdown. DA, dorsal aorta. PCV, posterior cardinal vein.


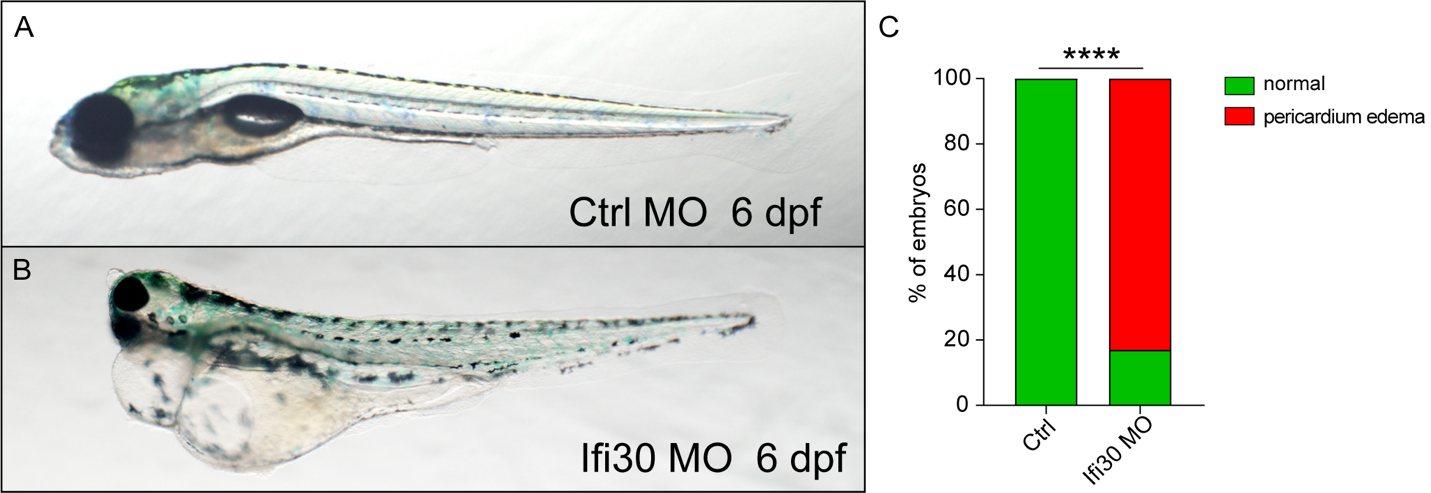


**Supplementary Figure 7.** **Ifi30 morphants exhibited pericardium edema at 6 days post fertilization (dpf) (A and B). (C)** The incidence of normal and pericardium edema phenotype ﻿in control *Tg(fli1ep:EGFP-CAAX)^ntu666^* embryos (n=20), and embryos injected with Ifi30 MO (n=63).

## Supplementary Table

﻿**Supplemental Table 1.** **Primers for ISH probe synthesis and RT-PCR.**

| *ifi30*-probe-F | 5’-GCCCAGGATGCAGAATGTTTC-3’ |
| --- | --- |
| *ifi30*-probe-R | 5’-GTGCATCGGTTTTCACTGCAT-3’ |
| *bmp2b*-probe-F | 5’-GAAGAGGCTTTCGAGGCACT-3’ |
| *bmp2b*-probe-R | 5’-GAAGGGAACGACTGACCCTC-3’ |
| *bmp2a*-probe-F | 5’-GCGCTGAAACAAGTGGATGT-3’ |
| *bmp2a*-probe-R | 5’-GAGGTGTCATTGTGGAGTGGT-3’ |
| *dab2*-probe-F | 5’-CAGAGGGCCAGCATCAGTTT-3’ |
| *dab2*-probe-R | 5’-CGTCGCTGAAGGGATCTGAA-3’ |
| *l-plastin* -probe-F | 5’-GACTCCTGTGGCAGGTCATC-3’ |
| *l-plastin* -probe-R | 5’-GTTGAGTTTGGGGTTTCCGC-3’ |
| *ef1α*-RT-F | 5’-GTGACCTTTGCCCCTGCTAA-3’ |
| *ef1α*-RT-R | 5’-CTCCAGCCACATTACCACGA-3’ |
| *kdrl*-RT-F | 5’-CGTTATCCTTGAGACGCAGATG-3’ |
| *kdrl*-RT-R | 5’-AATTCTGCGTTATCCACCCTGG-3’ |
| *fli1a*-RT-F | 5’-ATGAGAAATGTGCTCCGCCT-3’ |
| *fli1a*-RT-R | 5’-AAACATGGCCGTGTCGATCT-3’ |
| *dll4*-RT-F | 5’-CTCTGGCCGTTGGCTTAGT-3’ |
| *dll4*-RT-R | 5’-GTGTTCTTCAGCTGGGAGGT-3’ |
| *ifi30*-RT-F | 5’-GTTCGGCTTTAACCTGTGCG-3’ |
| *ifi30*-RT-R | 5’-CATGCATTGTTCCAGCACCC-3’ |

## Supplementary Movies

**Movie S1.** CVP development in control-MO injected *Tg(fli1ep:EGFP-CAAX)^ntu666^* transgenic embryos from 30 to 36 hpf.

**Movie S2.** CVP development in Ifi30-MO injected *Tg(fli1ep:EGFP-CAAX)^ntu666^* transgenic embryos from 30 to 36 hpf.
